# ‘Everyone assumes a man to be quite strong’: Men, masculinity and rheumatoid arthritis: A case‐study approach

**DOI:** 10.1111/1467-9566.12628

**Published:** 2017-10-15

**Authors:** Caroline Flurey, Alan White, Karen Rodham, John Kirwan, Robert Noddings, Sarah Hewlett

**Affiliations:** ^1^ Academic Rheumatology University of the West of England Bristol UK; ^2^ Centre for Men's Health, Faculty of Health & Social Sciences Leeds Beckett University Leeds UK; ^3^ Psychology, sports and exercise Staffordshire University UK; ^4^ Rheumatology University of Bristol UK

**Keywords:** men's health, masculinity, identity, rheumatology, rheumatoid arthritis, qualitative

## Abstract

Current literature has overlooked the impact of chronic illness on masculine identity. We therefore aimed to investigate the impact of rheumatoid arthritis (a long term condition, affecting more women than men) on masculine identity. Six focus groups with 22 men with rheumatoid arthritis (RA) (data reported elsewhere) followed by five one‐to‐one interviews with men (English, mean age: 59 years) sampled to reflect a heterogeneous experience of life with RA based on knowledge gained from the focus groups. Transcripts were analysed using thematic analysis and are presented as individual case studies. Whilst the case studies provide five distinct experiences, common themes can be drawn across them, such as the importance of paid work. The men needed to renegotiate their masculine identity to deal with their RA. Two dealt with this by pushing through pain to retain masculine activities, two replaced masculine roles they could no longer do with other roles, and one rejected masculinity completely. Men with long term conditions may need to re‐write their masculinity scripts to enable them to accept and adapt to their condition. However, some men struggle with this, which should be taken into consideration when designing self‐management services for men with long term conditions.

## Introduction

Postmodern theories of masculinities suggest masculine identity is socially constructed, and therefore propose that gender norms are dictated by social influences rather than biological differences (Addis and Mahalik 2003). Multiple masculinities exist and at different times and places some ways of being a man are culturally more desirable, which has been conceptualised as hegemonic masculinity (Connell 2005). Hegemonic masculinity is the ‘current most honoured way of being a man’, against which men position themselves (Connell and Messerschmidt 2005: 832).

From a young age boys, and as they are grown, men, learn to present their masculine selves in situationally appropriate ways (Frosh *et al*. 2002), thus they are able to emphasise different aspects of the hegemonic ideal enabling them to construct effective manhood acts (Schrock and Schwalbe 2009). This ability to alter displays of masculinities to fit different circumstances encountered across the life course (Spector‐Mersel 2006) has been termed ‘the chameleonisation of masculinity’ (Ward 2015). It is therefore possible that men who live with a long‐term condition have needed to adapt their masculinity script to protect their masculine identity (Spector‐Mersel 2006). In other words, when masculinities are ‘radically disturbed’ by illness, men are prompted to reflect on their gendered beliefs and the practices of masculinity they engaged in prior to illness (O'Brien *et al*. 2007). Previous research investigating the relationship between masculinity and chronic health has predominantly focused on typically male diseases (e.g. coronary heart disease) or gender‐specific diseases (e.g. prostate cancer) (O'Brien *et al*. 2007). However, the impact on masculine identity of living with an incurable long term condition that affects more women than men and has to be self‐managed daily has not been previously explored in depth.

Rheumatoid arthritis (RA) is a chronic, progressive and systemic autoimmune disease, characterised by fluctuating symptoms such as swollen joints, pain and fatigue (Hill 2006, Newman *et al*. 1995). RA affects more women than men (approximately 30% of the RA population are male) (Humphreys *et al*. 2013) and is thought to take a different course in men compared to women (Forslind and Hafstrom *et al*. 2007).

There is a paucity of literature exploring the impact of RA on men and their masculine identity nor how they manage this impact. A comprehensive literature review (Flurey *et al*. 2015) found that whilst there was no consensus on whether gender impacted on ability to cope with or self‐manage RA, there was a gender difference in coping styles.

It is likely that men with RA would have a different experience of living with and managing their RA to women, it is therefore of note that the majority of qualitative studies exploring the lived experience or self‐management of RA have been conducted either solely or predominantly with female participants: ranging from 78 per cent to 100 per cent female (Sanderson *et al*. 2011, Plach *et al*. 2004, Stenström *et al*. 1993) and only two qualitative studies have focused solely on men. One (Lack *et al*. 2011) recruited patients from only one UK Hospital and did not explore coping strategies. The other (Beaton *et al*. 2012) was conducted with US war veterans, who may have very different experiences of life to the general population and did not explore masculine identity.

The current study aimed to explore in depth the experiences of male RA patients in the context of their individual lives, the impact this has on their masculine identity; and how they are currently coping with and managing their RA.

## Patients and methods

### Study design

Focus groups are useful for examining opinions and beliefs about an issue, whilst individual interviews are effective in exploring personal experiences (Molzahn *et al*. 2005) and their combination can provide complementary views of a studied phenomenon (Lambert and Loiselle 2008). To explore both the breadth and depth of men's experiences of living with RA and its impact on their masculine identity, focus groups were conducted with men with RA, and then a sub‐set of the focus group participants were invited to take part in individual interviews (data published elsewhere, Flurey *et al*. 2016)).

The current paper focuses on five men who took part in the individual interviews as case studies to understand the issue of being a man with RA and the impact on masculine identity (Stake 1995).

Case studies allow the development of understanding and insight through in‐depth examination of a few individual lives, which are of interest for both their uniqueness and their commonality (Stake 1995). We sought to understand individual experiences based on each individual's account in the focus groups; their subsequent individual interview; and in the context of their clinical and societal situation.

### Participant selection and recruitment

Male patients with RA (Arnett 1990) were invited to participate in focus groups by the first author (CF). Patients attending a rheumatology outpatient appointment at one of three NHS Trusts in England were purposively sampled to reflect a range of age, disease duration, disability and drug treatment. A subset of the focus group participants were then invited to participate in semi‐structured interviews, sampled to reflect a heterogeneous experience of life with RA, based on knowledge about the participants gained from the focus group discussions. Data saturation of new ideas was reached in the focus groups, thus no new participants were introduced for the interviews phase. Ethics approval was granted by the London‐Bromley Research Ethics Committee (ref. 13/LO/0852).

### Focus groups

The focus groups were facilitated by the first author (CF) with a sub‐set co‐facilitated by a male patient research partner (RN), lasted approximately two hours, were digitally recorded and transcribed verbatim (Flurey *et al*. 2016).

### Individual interviews

Topic guides were specific to the individual. Participants were asked to expand on areas that emerged in the focus groups as pertinent to their experience of RA and encouraged to raise any topics important to their experience not covered in the focus groups. Participants were given the option of being interviewed at home or in their hospital. The interviews lasted around one hour, were digitally recorded and transcribed verbatim.

### Analysis and interpretation

Data from the focus groups and interviews were analysed using inductive thematic analysis, a method for identifying, analysing and reporting patterns (themes) within data without trying to fit it into a pre‐existing coding frame (Braun and Clarke 2006). Data were analysed according to Braun and Clarkes's (2006) guidelines and managed using NVivo 8 (QSR International, Brisbane). Focus group data were analysed first, then interview data.

The first author (CF) analysed all transcripts, and a sample was independently analysed (Guba and Lincoln 1994, Sandelowski 1986) by three researchers (AW, KR, SH) and a male patient research partner (RN). Team discussions and comparison showed that they reached comparable conclusions to the first author (CF).

Once the individual data sets were analysed, data relating to each individual participant were compared and collated to produce individual case studies. The intention was to provide a credible and authentic account that offers an understanding of the experience of living and coping with RA as a man, within the context of each individual's circumstances (Stake 1995).

## Results

Six men who had taken part in the focus groups were invited to take part in a one‐to‐one interview. Only one man (75yrs; disease duration 17yrs) declined to take part. He did not provide a reason, but in the focus group he explained that he is not comfortable talking about himself and does not normally take part in research.

Three broad typologies of the impact of RA on masculine identity could be identified from the focus group data: ‘Retaining hegemonic ideals of masculinity’; ‘Trying to renegotiate masculinity’ and ‘Rejection of hegemonic masculinity’. The case studies of each of the five men who agreed to be interviewed will be presented within the appropriate typology. The man who declined to take part was considered as belonging to the ‘trying to renegotiate masculinity’ typology. Characteristics of each participant can be found in Table 1.

**Table 1:** Participant characteristics

Pseudonym	Age	Age at diagnosis	Disease duration	Employment status	Current or previous role	Retired pre– diagnosis?	Marital status
David	58	39	19	Full time	Mechanical engineering	No	Single: Never married
Mark	49	44	5	Full time	Plumbing and heating engineer	No	Married
Ron	60	43	17	Retired	Builder	No: early retirement^a^	Married
Peter	61	56	5	Retired	Banker	No: early retirement^a^	Married
Charles	69	67	2	Retired	Musician and dancer	Yes	Widowed

a Still working when they were diagnosed but eventually took early retirement due to their RA

### Typology 1: retaining hegemonic ideals of masculinity

Retaining hegemonic ideals of masculinity seemed to be important to many of the men in the focus groups, who tried to hold on to activities and roles valued by hegemonic masculinity (e.g. sports activities; paid work) that they took part in before being diagnosed with RA. Two men were interviewed to reflect this typology, one was single (David), the other was married and saw being the breadwinner as part of his masculine role (Mark).

David is a 58 year old man, who has ‘had [RA] now for 20 years […] when I had it, I was still quite a fit young man’. David works full time in a mechanical engineering role and lives alone. Mark is a 49 year old man, who has had RA for five years, he is self‐employed and still works full time: ‘I'm a plumbing and heating engineer, I've got to use my hands’.

Both Mark and David chose to be interviewed in their local hospitals, which could have been due to the convenience of coming straight after work (David) or in a lunch break (Mark). However, it may also be that choosing to separate discussing their RA from the rest of their lives reduces the impact on their masculine identity.

This potential separation of identity is reinforced by David's lack of disclosure of his condition to either his employer: ‘To be honest with you, they [employer] don't even know’ or his friends and family: ‘I don't make a big point of it. Er a couple of me friends know about it’. He explained that this was because most people either ‘don't understand anyway’ or forget, due to the invisible nature of the condition:

‘There's nothing about you that looks different. Even close friends forget about it at times.’

Mark also avoids talking about his RA due to the lack of understanding from friends, who make vicious jokes. Humour can be used to subvert the power and influence of others and to challenge power inequalities. In this way humour can be used as a weapon to be hurtful (Williams 2009). It is possible that this is what is occurring in the following example:

They go, ‘Oh yeah, same old injury, you might as well, you might as well go and slit your throat, you're always complaining you're ill or you can't play [skittles]’ but it's not, it's physically if I held the ball in my hand, then my elbow would probably hurt. So then I don't do it and they go, ‘It's just an excuse’.

By attempting to construct their identity as a person with RA separately to their masculine identity it is possible that David and Mark may be able to avoid being seen as ‘other’ and thus protect their masculine self‐image.

Both David and Mark have retained physically demanding leisure activities, which seem to contribute to their self‐identity. David rides a motorbike, and arrived for both the focus group and the interview in his biker clothes. He highlighted that his abilities and stamina used to be superior to his current abilities: ‘I used to ride around all over the place, years ago, don't so much now. I wouldn't have thought nothing of doing 200 miles’. His identity as a biker seems particularly important to him, and he told the same story in both the focus group and the interview of having been left behind by his friends due to a flare‐up of his RA. This may have been particularly salient to him as he was forced to depart from hegemonic ideals of stoicism and competitiveness, which seem to be valued by this group:

Just couldn't go out [of] the hotel. I was so bad I just had to sit in the hotel, and there was nothing more frustrating for me, and I was sat at the hotel and all my mates was whizzing round Douglas prom[enade] on their bikes. Oh, I was gutted.

Whilst for Mark, retaining his identity as a fit, sporty man seems important to him (‘exercise is the thing’) and helps him manage his RA (‘I try and keep active because if I sit around on a Saturday or Sunday I seize up’). He cycles often (‘there's no impact on your body’) and goes skiing ‘every year’ and explains that this seems to help his RA:

I have just been skiing for a week and before I went […] everything was hurting and within two days of skiing everything loosened up.

He is aware that his ‘stamina levels [have] dropped dramatically’ and explains that ‘I would go blazing away all day long, I can't do that anymore’. However, he has still retained his competitive nature: ‘My wife can't keep up with me, but trying to keep up with my son, who is 21 […] he is hard to keep up with’. This need to retain sporting prowess is a highly valued hegemonic ideal, which is thought to excuse men from the need to participate in other masculine behaviours (Connell 2005).

It seemed important to both of them to speak highly of their level of pre‐RA ability, which may be a way of reinforcing their masculine identity, and emphasising that any loss of ability is due to their RA and not a lack of masculinity.

Remaining in paid employment is also particularly important to both Mark and David. For David this seems to form part of his self‐sufficient identity and he was adamant that he would not give up work due to RA: ‘the Doctor, he wanted to sign me off, I said ‘no’’. Working provides David with structure to his day, a goal to aim for: ‘I had to get up every day to go to work and it gave me a target, else I'd have just stayed in bed […] It kept me going’ and something to feel proud of (‘you do feel a bit prouder’), due to the way he thinks others perceive him:

He [Rheumatologist] also had respect for me that I wasn't just prepared to throw the towel in.

Mark holds a strong masculine identity as the breadwinner (‘We've got a living to make so we've got to keep working and taking painkillers’). He seems particularly aware of his financial responsibilities to his children: ‘My daughter was in University and my son was in school, you haven't really got a lot of choice but to carry on, even to go through the pain’. He describes how he has previously continued working despite experiencing an intense amount of pain:

I was trying to work, I was in that much pain in my hands, I had to go to work and I was crying in pain when I used the tools that much, but you still had to do it every day, day in and day out. I think you could probably overdose yourself with painkillers, but I didn't.

Both Mark and David seem to have enough autonomy in their roles that they can manage to work around their RA. Whilst David has control over his hours and the tasks he undertakes (‘I can plan my day’), Mark is self‐employed and alters his work to accommodate his RA:

I am getting into more servicing of boilers and maintenance, because obviously the installs are getting harder and harder.

He explained that ‘every day [the job] is getting harder […] it is a manual job but my strength is probably down 50 per cent to what it was’ and there are things that he can no longer do:

I have to get other people now to lift heavy things, or where I used to lift a heavy boiler off the wall, I can't do it anymore, it's impossible.

Productivity and breadwinning are defining features of hegemonic masculinity, reinforced by societal expectations and the media (Vigorito and Curry 1998). For men in relationships occupational status and income are important for eliciting deference from their partners (Schrock and Schwalbe 2009), which may be even more important in the face of illness as a threat to masculinity. Men from working class backgrounds may distance themselves from intellectual work (seen as feminine) and embrace physical work (seen as masculine) to produce self‐protective displays of toughness (Fine *et al*. 1997). It is therefore possible that remaining in paid physical work is key to Mark and David positioning themselves as masculine.

In the focus group, both Mark and David seemed comfortable speaking in front of others and were often the first people to answer my (CF) questions in their respective groups. David reflected on this during our interview, which seems to explain the role he took on in the focus group:

It needs one to fire the others off. Erm if they're all a bit restrained you ain't going to get much information … you've got to get a bit of a leader […] Got to get one person that's you know forefront in their views, and happy to talk about it, then that brings the others on.

This way of positioning himself in the group may provide David with a justification for being in the group. This allows him to protect his masculine identity by fulfilling the masculine script that men should give to rather than receive something from a group (Seymour‐Smith 2008).

Both David and Mark easily opened up about their limitations and how their frustration had previously driven them to tears:

I was in tears on the garage floor, because I'd got a drill and I couldn't get up off the floor. (David)

When it's bad, I mean you're crying because you can't physically use your hands. (Mark)

Crying is assumed to violate masculine norms regarding the importance of emotional control (Wong and Rochlen 2005). However, the expression of ‘manly emotion’ can be permissible as it conveys a degree of self‐control that remains consistent with hegemonic masculinity (Warner and Shields 2007). Their disclosure of being *‘in tears’* fits the description of ‘manly emotion’ as it relates to the experience of frustration with physical limitations that the majority of the group are likely to have experienced. However, in the interview setting, David was able to open up about more vulnerable feelings of helplessness:

You end up in tears because you just can't understand what's going on […] that was why it was so downing because it come on so fast, you didn't know where it was going to stop.

He was reluctant to use the word ‘depressed’ and settled on ‘it's aggravation rather than depressed’, perhaps because this more acceptable term retains his self‐image of being ‘mentally strong’.

Interestingly, both Mark and David use the third person narrative (e.g. David: ‘you end up in tears’) when discussing their distress, which could be a way of distancing themselves from difficult emotions.

Mark and David's narratives indicate that it is important to them to retain their hegemonic masculine ideals and their pre‐RA masculine activities, whilst acknowledging that their abilities have declined. Their newly adopted masculinity script enabled them to portray an outward image of managing well with their condition and continue their pre‐RA lifestyle. However, RA was a challenge to their masculinity, which they deal with by compartmentalising their identity as a man with RA and by attempting to hide this impact from other people. Thus, they may be skilfully wearing what Pollack (1998) termed the ‘mask of masculinity’ to hide feelings of vulnerability, powerlessness or isolation.

### Typology 2: ‘trying to renegotiate masculinity’

Men who were no longer able to take part in certain masculine activities, which had been important to them before being diagnosed with RA had to deal with this loss to their masculine identity. Two men were interviewed to capture this typology, one of whom was learning to replace previous masculine activities with re‐defined masculine activities (Ron), and the other who was struggling to cope with this loss (Peter).

Ron is a 60 year old man, who has had RA for ‘about 20 years now’, whilst Peter is a 61 year old man, who has ‘had RA for about 5 years’. Both Peter and Ron chose to be interviewed in their own homes, which may be due to the convenience of the researcher visiting them as they both have a more severe impact of RA on their mobility than David and Mark. However, it may also indicate that they see RA as such a large part of their lives that their home cannot be separated from their experience.

A big loss to both Ron and Peter was having to take early retirement due to their RA. Peter explained this had a ‘massive impact’ on him and led to him being diagnosed with depression. In the focus group he explained: ‘I had a job that I enjoyed […] Took a package [severance pay] and came out’. In the interview Peter explained that it wasn't necessarily the physical ability to do his job that was the problem, but the ‘psychological aspect’ (‘the stress and strain’). He doesn't feel that his employer supported him ‘as [he] would like to have been supported’ to remain working (‘I worked for banks […] just really want to make money don't they?’). This seems particularly difficult for him as he ‘loved what [he] did’ and seemed to have held a lot of responsibility in the job.

Before being diagnosed with RA Ron was ‘in the building trade’. He explains that the ‘hands on’ work ‘became a struggle’. However, when his RA was under control with medication ‘it made a big difference’ and Ron expected to be able to return to working full time: ‘You didn't like to say ‘no’ to anyone so you were working 12 hours a day, after being out of work for three years. Then within about three and a half months it all came back and that was hard’. He therefore stopped working due to his RA, and didn't feel able to take on a non‐manual job (‘The education wasn't there, the brains weren't there […] That's why I was always manual like, I couldn't go on a computer’).

The loss of full time employment for both Peter and Ron seems to have impacted strongly on their identity as working men. Ron holds the hegemonically masculine view that the man should be the breadwinner (‘He is the head of the home, when he is a family man’) and so felt uncomfortable stopping working (‘a guilt complex that you're not earning your money’). He also explained that ‘a man is happier working […] a man can thrive on work […] the rewards of work is not just money, it's the satisfaction of a job well done’. The use of the third person narrative seems to be used to normalise his views of traditional male roles and expectations of how men should think and behave.

Being able to complete tasks around the home was important to Ron: ‘consequently that [gardening work] is troubling my legs, but the bonus is you achieve something […] and when you achieve something, that gives you a boost’. As men get older they remain involved in activities that define them as men, even if these activities take on a different form, thus a ‘busy ethic’ can replace a ‘work ethic’ (Thompson and Whearty 2004) and in this way it seems that Ron is able to renegotiate his masculine identity from his role as the ‘breadwinner’ to being useful in the home.

However, Peter still seems to be struggling to find new ways to keep himself busy. Peter lives in a quiet cul‐de‐sac and explained that he moved to this area with his wife to be closer to his daughter and grandson. However, for Peter, moving house has meant that he has lost his social support network (‘The only people that I know really down here are my daughter and her husband’), which is not easy to rebuild due to his difficulty leaving the house (‘I don't get out a lot’).

Peter will often pay someone to do the things he can no longer do around the house. This supports the social exchange theory (Jackson *et al*. 2012), which proposes that human relationships rely on negotiated exchanges based on a cost‐benefit analysis by both parties. It is likely that in his previous social network Peter would have built up ‘credit’ and thus feel more comfortable asking for favours. However, in his new home Peter no longer has a familiar network around him and therefore is concerned that if he accepted a favour he would not be able to repay it in kind, thus he feels more comfortable paying for any services he receives:

It's just easier to pay someone and fortunately at the moment I am in a position where I can afford to pay someone […] if you can afford it it's better to pay people isn't it rather than think ‘am I getting on their nerves by asking too much?’

It is possible that through acting as an employer rather than a person accepting a favour that this is an alternative method of renegotiating masculine identity.

Peter explained that due to his RA he is ‘pretty limited in what [he] can do’. He particularly feels he is missing out on being able to play with his grandson: ‘There's things I can't do with him that I'd like to. I can't pick him up’, which is particularly difficult because ‘he's at the age where you'd like to do more throwing around, picking him up and playing with him’. This loss of the way that hegemonic masculinity would dictate a man should behave with a male child is particularly pertinent when considered in the context of research that suggests positive interactions between grandfathers and grandsons are related to fewer depressive symptoms and positive affect in older men (Bates and Taylor 2012). Whilst some grandfathers choose to engage in potentially risky embodiments of masculinity such as physical play, others emphasise their wisdom and experience (Tarrant 2013). At 61 years Peter is still a relatively young grandfather and seems to hold expectations of himself to be able to perform masculinities that he used to enact as a father, thus this discrepancy may be acting as a threat to his masculinity.

Ron and Peter's narratives indicate that the loss of paid work through illness has a strong and lasting impact on masculine identity. It is suggested that retirement can prompt a crisis of masculine identity due to the estrangement from masculine assumptions (Oliffe *et al*. 2013). However, it is possible that this crisis is further pronounced by the need for early retirement due to illness, with a stepped reduction often not being possible, and often with little choice or control. It therefore seems important to learn to renegotiate masculinity by adopting new masculine tasks, but this can be a difficult and slow process.

### Typology 3: ‘rejection of hegemonic masculinity’

A more unusual experience of living with RA amongst those in the focus groups was the experience of being comfortable enough within one's identity to reject or be unconcerned with the need to behave in a hegemonically masculine way. One man was interviewed to capture this typology (Charles)

Charles is a 69 year old man (‘celebrating my seventieth birthday soon’), who lives alone and was retired prior to his diagnosis of RA two years ago. My (CF) first impression of Charles was of a softly spoken, polite man, who asked for permission to speak (‘Can I come in on this one?’). He seemed comfortable being himself in the all‐male focus group (‘no problem presenting myself’), and talked easily about the activities he enjoys, which may not be considered traditionally masculine according to westernised hegemonic ideals (Connell 2005): ‘I've always been active with dancing, playing badminton and playing musical instruments’.

Charles opted to be interviewed in his own home. The first thing I noticed as I walked in was his immersion in music, with musical instruments and sheet music covering every surface. It was therefore not surprising that Charles’ relationship with music characterised his experience of RA and seemed to define his identity.

Charles explained how his RA has impacted on his ability to play his mandolin (‘it was a great loss to me not to be able to play certain tunes’) and he discussed the impact this has on being able to help his grandson:

especially when I'm trying to show my grandson some finger‐work or, well yeah it can be, erm, it can be disheartening because the tunes I played for hundreds of times and I now can't manage and if I play them slowly they're not effective, I mean you can't play a jig in slow time. So that is my current loss.

Despite this activity not conforming to the hegemonic standards of physical or rough games that a grandfather ‘should’ engage in with his grandson, it is important to note that Charles still misses being able to pass on his skills and knowledge to the younger generation.

Charles seems to have a strong identity as a musician and a dancer (‘I think your life should include dancing’), thus he seems to be finding ways of retaining this identity by adapting to his RA:

I'm in a group, a musical group, but in order to stay with the group I have to reduce my repertoire to the tunes I can manage.

Charles argued that it is easier to deal with RA when retired: ‘not having to work gives you the time to cope’. He seems satisfied with the position he is in at his age: ‘We've got television, music, reading, that's fine for a seventy year old. And my family nearby, so I'm doing quite well’.

Charles does not come across as a ‘typical man’ within the confines of our understanding of traditional hegemonic heterosexual masculinity. His soft voice, calm demeanour, and interest in activities that do not conform to hegemonic ideals of masculinity, could be considered as ‘hiding in the shadow of masculinity’ (Heasley 2005). Whilst not conforming to masculine ideals, men ‘in the shadow of masculinity’ know enough about hetero‐normative expectations to be able to fit in (or ‘pass’) with a group of men (Buchbinder 2010).

However, a further explanation is that Charles may be so comfortable in his own identity that he rejects the need to behave in hegemonically masculine ways, or feels so comfortable within his masculinity that he doesn't feel the need to ‘prove himself’ as a man and therefore can engage in activities such as dancing without his masculinity being threatened. This ability to reject the demands of hegemonic masculinity seems to be both physically and psychologically protective for him: ‘All I'm doing is taking pills and enjoying life’.

### Representations of masculinity

Previous research with school age boys suggests that they present their masculinity differently depending on the situation (Frosh *et al*. 2002). The authors reported that boys were ‘serious and softer’ in the interviews, yet ‘free and funny’ in the focus groups. This is supported by our findings that many of the men were able to expand on more sensitive issues in the interviews than focus groups. Further, in the focus groups men used more humour and ‘banter’ both as a way of bonding with each other in the group, and to deal with difficult issues:

Trying to put a shelf up because you don't trust your wife with the electric drill, things like that [all laugh]. (Mike 60yrs, focus group participant)

I have to laugh at myself, levering myself out of the car. (David)

Whilst in the focus groups it seemed important for many of the men to act as ‘one of the lads’ and in doing so the female researcher (CF) could be treated as an outsider who they (as a group) could explain their experience to. Nevertheless traditional values of the way ‘real men’ should treat women often made them censor their views, to protect the researcher from being insulted:

I'd always prefer a woman [physiotherapist]. I just imagine I can carry it a stage further, I'm on the beach… (Charles)

I do enjoy working, I mean, I enjoy being with the lads and everything else. It's all, all fellows, I'm with, no women. But it's a, it's a very heavy job, so, you know, women couldn't do that. I suppose they could but. I don't mean to be… (Will 61yrs, focus group participant)

Further, the group steered individual men away from topics seen as problematic such as Ron discussing his Christian faith (‘I didn't think I was coming here to be converted today’ Edward, 60yrs, focus group participant) and Peter discussing his depression:

I think most males will try and fight the depression, they will, within reason. To be honest I'm running a business and I haven't got a choice. (Mark)

However, in the interviews the men seemed to set themselves apart from behaviours that they felt were stereotypically masculine or inappropriate:

Men'll say ‘oh no I don't want to go to that group because all you do is talk about your problems. (Ron)

I remember him [participant] saying he nearly punched him [health care professional who hurt him] and I thought ‘oh dear, that's dreadful’, you know when people are there trying to help. (Charles)

Thus, in the more private setting of the interview it seemed men were more comfortable departing from expectations to behave in a way that conforms to hegemonic masculinity.

Frosh *et al*. (2002) proposed that neither of these interactions are more ‘authentic’ than the other but that each setting draws out a different manifestation of masculine identity construction or different ways of ‘doing boy’. Our findings suggest that this construction of multiple masculine identities depending on the setting still seems to remain in adulthood.

## Discussion

These case studies provide three typologies for explaining the impact of RA on masculine identity: ‘Retaining hegemonic ideals of masculinity’; ‘Trying to renegotiate masculinity’ and ‘Rejection of hegemonic masculinity’. Despite the heterogeneity of these experiences some important commonalities can be drawn across these case studies that may improve our understanding of men with long term conditions, such as rheumatoid arthritis.

The importance of paid employment was a theme that arose across all five case studies. This was highlighted through the fierce determination of both Mark and David to stay in work; the loss experienced by Peter and Ron through having given up work; and by Charles explaining that his RA is easier to manage as he was diagnosed post‐retirement. This supports previous findings in RA that men in paid work report significantly lower levels of emotional distress than women in paid work, but that men who have stopped working report significantly higher distress than women who have stopped working (Fifield *et al*. 1996). This may be due to men having difficulty relinquishing their role as the ‘breadwinner’ (Stamm *et al*. 2010), which reflects findings in other conditions such as coronary heart disease (O'Brien *et al*. 2007). The majority of the men are or were engaged in paid work, which enables or enabled them to enact masculine ideals such as financial dominance, being in a position of authority, or displaying physical strength (Connell 2005). However, Charles’ role as a musician and dancer did not fulfil hegemonic ideals of masculinity, and therefore although an important part of his identity, this may not have contributed to the way he positions himself as a man. Although retired, Charles continues to play music, thus his expertise in this may be more important in his construction of masculinity than paid work.

Previous RA research has suggested the need for men to ‘depart from their masculinities’ to allow them to adjust and accept their RA (Lack *et al*. 2011). However, these data suggest renegotiation of rather than departure from masculinity. For Mark and David retaining their masculine identity as strong, working men, able to ‘keep up with the boys’ in their social life seemed particularly important. They both dealt with their RA by carrying on despite the pain, making adjustments to their working life and continuing to take part in masculine activities such as sports and motorcycling. Peter and Ron, were in a renegotiation process (Thompson and Whearty 2004) involving trying to find ways of holding onto their masculine identity despite a reduction in abilities. Both have struggled to accept this and are still adjusting to their limitations. This need to renegotiate the meaning of masculinity in the face of illness reflects findings within other conditions such as prostate cancer (Maliski *et al*. 2008). In contrast, Charles deals with his RA by staying positive and focusing on pleasurable events in the future, he doesn't seem to allow RA to impact on his life, and RA does not appear to have a clear impact on his masculine identity. This could be due to more acceptance of a slower pace of life due to his older age and being retired at disease onset. However, he has also experienced losses of activities he previously enjoyed, thus it is possible that other role identities may be more salient to him than his gender identity, or that he feels so comfortable in his masculinity that he does not allow RA to impact on this.

The men who took part in this study were willing to talk about their experiences of RA in both a focus group and a one‐to‐one interview setting, adding further weight to the evidence that suggests generalisations about men being ‘strong and silent’ can be misleading. Similar results have been found when interviewing men with depression (Emslie *et al*. 2006) and prostate cancer (Oliffe and Mróz 2005). Thus, given the right supportive environment (preferably one with a practical focus) and permission to talk about sensitive issues men are willing to talk about not only their physical but also their emotional experiences (Galdas *et al*. 2014). Others suggest that men are more likely to attend support services as a volunteer (Milligan *et al*. 2013). Thus, the research interview setting may be seen as task‐orientated volunteering, and men may find it easier to justify speaking openly as a way of furthering research rather than for their own benefit.

It is of note that these men seemed more able to open up about more sensitive issues in the interviews than the focus groups and portrayed their masculinity differently in both, supporting previous findings with school age boys (Frosh *et al*. 2002). This may be due to the interviews being the second meeting and thus having gained more trust in the researcher. It may also be due to the one‐to‐one nature of the interviews meaning the men did not feel the need to position themselves as they might within a group of other men. The findings from this study indicate that this changing construction of masculine identity is not something men grow out of, it is likely that this (re)negotiation of masculine identity is constant and often unconscious amongst men as they navigate different relationships and attempt to ‘pass’ as ‘one of the boys’ (Buchbinder 2010).

Our study consists of a group of men with RA who were willing to take part in focus groups and follow‐up one‐to‐one interviews. It is therefore possible that we are missing the voices of the ‘strong and silent’ men (O'Brien *et al*. 2005). Indeed, the one man who declined to take part in the interview phase was invited due to his perspective as a ‘strong and silent’ man. However, his voice has been included elsewhere (Flurey *et al*. 2016). The strengths are that this study sampled for a range of age, disease duration and disability; from seven consultants across three NHS trusts; and then further sampled based on knowledge gained from the focus groups about these men's experiences and coping styles, thus providing five distinct and heterogeneous disease experiences. Further, a male patient research partner contributed to the study design, data collection, and interpretation (RN).

These data provide important information on the experiences and coping styles of men with RA and the impact this has on their masculine identities. These novel data about the impact of a long term condition (RA) on masculine identity indicate that to incorporate RA into masculine identity it is necessary for masculinity scripts to be re‐written, which some men find challenging. These five distinct case studies highlight the different coping strategies and support needs that should be considered when designing support and self‐management services for men with long term conditions.

Further research should investigate whether there are common issues relevant to men with similar characteristics. This is now being addressed through a Q Methodology study informed by these findings, to investigate whether men can be grouped according to the impact of RA and their coping styles, which would have the potential to inform the design of a support intervention. Further research should also investigate the generalisability of these findings, which will enable an informed judgement of whether there is a clinical need to provide services tailored towards the potentially different needs of men.
